# Monotonic Optimization of Dataflow Buffer Sizes

**DOI:** 10.1007/s11265-018-1415-2

**Published:** 2018-10-23

**Authors:** Martijn Hendriks, Hadi Alizadeh Ara, Marc Geilen, Twan Basten, Ruben Guerra Marin, Rob de Jong, Steven van der Vlugt

**Affiliations:** 1 ESI (TNO), Eindhoven, The Netherlands; 2 Eindhoven University of Technology, Eindhoven, The Netherlands; 3 Philips Healthcare, Best, The Netherlands; 4 TOPIC Embedded Systems, Best, The Netherlands

**Keywords:** Monotonic optimization, Cyclo-static dataflow, Throughput, Buffer size

## Abstract

Many high data-rate video-processing applications are subject to a trade-off between throughput and the sizes of buffers in the system (the storage distribution). These applications have strict requirements with respect to throughput as this directly relates to the functional correctness. Furthermore, the size of the storage distribution relates to resource usage which should be minimized in many practical cases. The computation kernels of high data-rate video-processing applications can often be specified by cyclo-static dataflow graphs. We therefore study the problem of minimization of the total (weighted) size of the storage distribution under a throughput constraint for cyclo-static dataflow graphs. By combining ideas from the area of monotonic optimization with the causal dependency analysis from a state-of-the-art storage optimization approach, we create an algorithm that scales better than the state-of-the-art approach. Our algorithm can provide a solution and a bound on the suboptimality of this solution at any time, and it iteratively improves this until the optimal solution is found. We evaluate our algorithm using several models from the literature, and on models of a high data-rate video-processing application from the healthcare domain. Our experiments show performance increases up to several orders of magnitude.

## Introduction

Many high data-rate video processing applications have strict requirements on throughput as it affects the (visual) quality. It may even affect safety, as is the case for the medical video-processing application in the image-guided therapy domain that has motivated our work. Often, these types of applications are subject to a trade-off between throughput and the sizes of the buffers (the *storage distribution* from now on). Since buffer space uses expensive or scarce resources, one of the key design questions for these applications is how to minimize the storage distribution without violating the throughput constraint. We approach this problem using model-based design, and model and analyze the application using the cyclo-static dataflow (CSDF) formalism [5]. This formalism is a member of the dataflow family [6] and is suitable for modeling a broad class of streaming, parallel applications with cyclically changing behavior and finite buffers such as our video-processing applications. This model-based approach has the advantage that the analysis of the model usually is much faster than experimentation on a prototype. For instance, our driver case has an FPGA as implementation target. The hardware implementation step in the process takes several hours. Using a storage-distribution minimization algorithm with an analysis step that takes several hours clearly is infeasible for even small search spaces.

Efficient methods exist to compute the throughput of a CSDF graph with a given storage distribution. The problem that we consider in this article is to minimize the size of the storage distribution under a throughput constraint. In general, this problem is NP-hard [8], and we therefore present an *anytime* algorithm. The algorithm first tries to quickly find an initial storage distribution that realizes the throughput constraint. Then it iteratively improves the storage distribution. During this process, the algorithm provides an upper bound on the difference between the size of the currently best storage distribution and the size of the (unknown) minimal storage distribution. This can be a useful feature because if the user has no patience to wait for a real minimum storage distribution (finding one can take long due to the NP-hardness), he can terminate the algorithm and still have a feasible storage distribution and an estimation of the quality of this solution.

### Contribution

In this work we combine principles from monotonic optimization [12, 13] and the concept of knee points of [7] with the causal dependency analysis from [10, 11]. This results in an algorithm that minimizes the storage distribution in CSDF graphs under a throughput constraint. This algorithm scales better than the state-of-the-art approach of [10, 11]. Our experiments show that the performance may be improved by several orders of magnitude. Furthermore, it is an anytime algorithm which is able to present at any moment (after the initialization phase and if it exists) a storage distribution that satisfies the throughput constraint and a bound on the suboptimality of this best solution so far. A secondary contribution is an elaboration of the concept of knee points that has been introduced in [7]. Knee points play a crucial role in our algorithm.

### Related work

Closely related work that adresses the problem of this article, optimization of the storage distribution under a throughput constraint for CSDF graphs, is the work of [3, 4, 10, 11, 15]. In [15], a fast approximation algorithm is proposed that over-estimates the size of the required storage distribution with an unknown factor. The work of [10, 11], on the other hand, presents an exact solution to a slightly more general problem than the problem of this article: [10, 11] compute the whole trade-off space, which can then be used to solve our problem. The work of [3, 4] is closely related to [15] and provides an approximate solution based on a relaxation of an integer-linear program. Our work is complementary to [10, 11] as it can be regarded as a fast heuristic to significantly prune the search space after which the exact method of [10, 11] is used to obtain the final solution. Our method can also be regarded as a domain-specific specialization of the domain-independent and generic monotonic optimization framework of [12, 13]. This framework to solve non-convex, but monotonic optimization problems, has succesfully been applied in the area of wireless communications [9, 14, 16], and we now introduce it in the dataflow domain. The key difference with the generic outer-polyblock approximation algorithm of [12, 13] is that we bound the optimal solutions from both the inside and the outside. This is similar to the approach of [7], which uses a constraint solver to build an approximation of the Pareto front of a multi-criteria optimization problem, using monotonicity implicitly. We use the concept of knee points of [7] to select a new point in the search space to explore, instead of using a binary search to compute the upper-boundary projection in the outer-polyblock approximation algorithm of [12, 13]. Furthermore, the fact that we limit the scope of the approach to CSDF allows us to take advantage of domain-specific properties and analysis methods, i.e., the causal dependency analysis of [10, 11], to make the search more efficient.

## Explanation of the Approach

**Figure 1:**
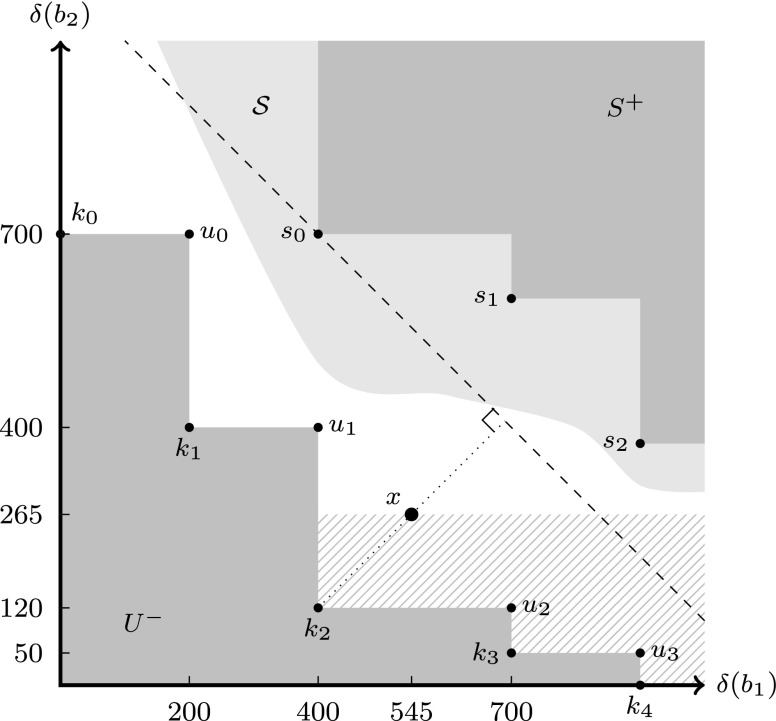
Sketch of the approach.

Let us consider a 2-dimensional buffer sizing problem modeled in CSDF with buffers *b*_1_ and *b*_2_. A *storage distribution* is a function $\delta : \{ b_{1}, b_{2} \} \to \mathbb {N} \cup \{ \infty \}$ that gives the size of each buffer, measured in dataflow tokens. The set $\mathcal {S}$ of *feasible* storage distributions (those storage distributions that satisfy the throughput constraint) is shown in Fig. 1 (it appears to have a smooth boundary due to the scale of the figure). Of course, we do not know $\mathcal {S}$ beforehand, and computing whether a storage distribution is feasible by invoking a CSDF throughput analysis can be time consuming. Let us assume in this example that both buffers contain items of equal size, and that we want to minimize the size of the total storage distribution, i.e., the sum of the two buffer sizes.

Our approach is centered around monotonicity of throughput and buffer sizes in the CSDF formalism: increasing buffer sizes will not decrease throughput. This monotonicity allows us to efficiently represent and bound the search space. Suppose that we have analyzed four points in the storage distribution space *u*_0_ – *u*_3_ and that these resulted in a less than required throughput (they are infeasible). Figure 1 shows these points, and because of monotonicity we know that the area *U*^−^ does not contain any feasible points. In a similar way, we can build a view of feasible points. Suppose that *s*_0_ – *s*_2_ are points that we have analyzed and that have been shown to be feasible. The area *S*^+^ then only contains feasible points because of monotonicity. Because the total size of the storage distribution is also monotone, the best solution in *S*^+^ is one of the points *s*_0_ – *s*_2_, namely *s*_0_ with size 400 + 700 = 1100. This representation of *U*^−^ and *S*^+^ and the exploitation of monotonicity is closely related to the area of monotonic optimization [12, 13].

The second concept that we use are the special *knee pointsk*_0_ – *k*_4_ [7]. These are induced by *u*_0_ – *u*_3_ and are local minima in the sense that any feasible solution will have a size greater than the size of one of the knees (because of monotonicity). In particular, the knee points with the smallest size provide a lower bound on the size of any feasible solution. In this example, *k*_2_ has the smallest size of 400 + 120 = 520. This means that the optimal solution (at best the point (401,121)) has a size of at least 522 and at most 1100 (size of *s*_0_). We thus say that the maximal error Δ equals 1100 − 522 = 578. Given the state of knowledge determined by the set of feasible points *s*_0_ – *s*_2_ and the set of infeasible points *u*_0_ – *u*_3_, we select a new point to check for feasibility. This selection process is based on the knees and on the hyperplane of points with size equal to the best solution so far (the dashed line through *s*_0_): We select a point *x* = (545,265) halfway on the line segment between the hyperplane and a knee with the smallest size (*k*_2_ in this example). Intuitively, this is the area where most can be gained. By choosing the point *x* halfway between *k*_2_ and the hyperplane, we apply a multi-dimensional binary search. In the case that *x* is feasible, we extend *S*^+^ with the area to the right and above *x*. Furthermore, *x* improves on *s*_0_ and the maximal error Δ now equals 810 − 522 = 288, which is approximately half of the previous maximal error.

The third ingredient in our approach is the causal dependency analysis of [11], which we use to bound the search space even further. Throughput analysis of a CSDF graph can, in addition to the throughput, also provide the channels that have a so-called *storage dependency*. Intuitively, a channel creates a storage dependency if the progress of the data processing depends on freeing storage space in the buffer associated to that channel. From [11] it follows that throughput can only increase if the size of at least one channel with a storage dependency is increased. Now, suppose that the analysis of *x* shows that it is infeasible and that only buffer *b*_2_ has a storage dependency. This means that not increasing the size of buffer *b*_2_ from point *x* will never result in a feasible point. We therefore can extend the infeasible point *x* = (545,265) to (*∞*,265), resulting in a significant reduction in the search space: the area filled with the pattern is added to *U*^−^. The knee points *k*_2_, *k*_3_ and *k*_4_ are removed, and a new knee point *k*_5_ = (400,265) is added. This makes *k*_1_ = (400,200) the knee point with the smallest size in the new situation, and this reduces the maximal error Δ from 578 to 1100 − (401 + 201) = 498.

Iteration of these steps reduces the gap between *U*^−^ and *S*^+^ and also the maximal error Δ, and will eventually find the best feasible storage distribution.

## Cyclo-Static Dataflow Graphs

We briefly repeat existing definitions and results concerning CSDF graphs based on [11]. We let $\mathbb {N}_{0,\infty }$ denote the set $\mathbb {N} \cup \{0, \infty \}$. Let *P* be a set of *ports*, and let *rate* be a function that assigns a finite sequence (*r*_1_,*r*_2_,…,*r*_*n*_) of rates in $\mathbb {N}$ to each port (lengths of these sequences may differ among the ports). An *actor* is a tuple (*I*,*O*,*T*) consisting of *I* ⊆ *P* input ports, *O* ⊆ *P* output ports with *I* ∩ *O* = *∅*, and of *T* = (*t*_1_,*t*_2_,…,*t*_*n*_) execution times.

### **Definition 1** (CSDF graph)

A CSDF graph is a tuple (*A*,*C*) of a set of actors *A*, and a set of channels *C* ⊆ *P* × *P* such that (i) (*p*,*q*) ∈ *C* implies that *p* is an output port and that *q* is an input port, and (ii) all ports are connected to exactly one channel.

The initial state of a CSDF graph is determined by the initial token distribution, which assigns a number (possibly 0) of initial tokens to each channel.

**Figure 2:**
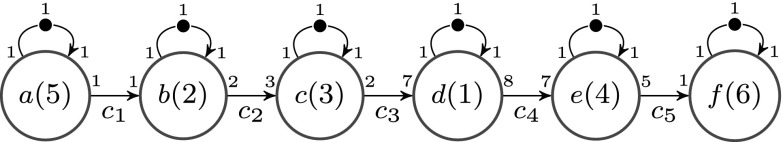
The CSDF graph for a sample-rate converter.

Consider, for instance, the CSDF graph in Fig. 2. It shows the graph of a sample-rate converter [1]. The nodes represent the actors and the edges represent the channels (ports are not explicitly shown). The execution time sequence is shown in the actor nodes. The numbers at the beginning and end of the edges show the rates. Note that all execution time and rate sequences have length one in this example (effectively turning this model into a Synchronous Dataflow (SDF) graph). Each self-loop has a single initial token which limits auto-concurrency of the actors.

Channels have an unbounded storage space in the semantics. As it is commonly done in literature, we model finite buffer space of channels *C*_*buf*_ ⊆ *C* by adding for each channel (*p*,*q*) ∈ *C*_*buf*_ from actor *a* ∈ *A* to actor *b* ∈ *A* a new channel (*p*_*δ*_,*q*_*δ*_) from *b* to *a* where *p*_*δ*_ and *q*_*δ*_ are new ports with *rate*(*p*_*δ*_) = *rate*(*q*) and *rate*(*q*_*δ*_) = *rate*(*p*). The number of initial tokens on (*p*_*δ*_,*q*_*δ*_) equals the storage space of the channel (*p*,*q*) minus the number of initial tokens on (*p*,*q*).

### **Definition 2** (Storage distribution)

Let (*A*,*C*) be a CSDF graph and let *C*_*buf*_ ⊆ *C* be a set of buffered channels. A storage distribution for *C*_*buf*_ is a function $\delta : C_{\mathit {buf}} \to \mathbb {N}_{0,\infty }$. We let (*A*_*δ*_,*C*_*δ*_) denote the CSDF graph with the additional channels that realize the storage constraints,^1^ and assume that it is strongly connected.^2^

**Figure 3:**
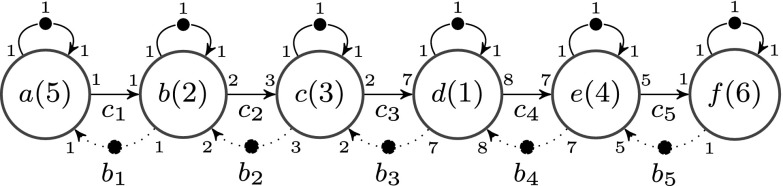
The CSDF graph for a sample rate converter with additional storage constraints.

Consider the CSDF graph (*A*,*C*) of Fig. 2, and let *C*_*buf*_ = {*c*_1_,*c*_2_,…,*c*_5_}. Consider the storage distribution *δ* such that *δ*(*c*_*i*_) = *b*_*i*_ for 1 ≤ *i* ≤ 5. Figure 3 shows (*A*_*δ*_,*C*_*δ*_). The dotted edges represent the special channels that model the storage constraints. For instance, the constraint on the channel *c*_1_ is modeled by the dotted channel from *b* to *a* with *b*_1_ initial tokens on it. This models that there can be at most *b*_1_ tokens in *c*_1_.

Let *δ* and *δ*^′^ be two storage distributions. We say that *δ* ≼ *δ*^′^ if and only if *δ*(*c*) ≤ *δ*^′^(*c*) for all *c* ∈ *C*_*buf*_. Since the tokens in different channels may represent data of different size we introduce a *cost* function $w : C_{\mathit {buf}} \to \mathbb {N}$ that assigns a (non-zero) cost to each buffer channel. The *cost* of a storage distribution *δ*, denoted by |*δ*|, then is ${\sum }_{c \in C_{\mathit {buf}}} w(c) \cdot \delta (c)$.

Throughput of a CSDF graph is a well-defined concept and algorithms exist to compute it (see [11]). For (*A*,*C*) we let $\xi (A,C) \in \mathbb {R}$ denote its throughput. A throughput constraint $\mathit {tc} \in \mathbb {R}$ on (*A*,*C*) gives a lower bound on the necessary throughput. We say that a storage distribution *δ* is *feasible* if and only if the throughput constraint is satisfied, i.e., *ξ*(*A*_*δ*_,*C*_*δ*_) ≥*tc*. A useful property of the CSDF formalism is that throughput, and thereby feasibility of storage distributions, is monotone with respect to buffer sizes.

### **Lemma 1**

[11] *Let* (*A*,*C*) *be a CSDF graph, and let* *δ,δ*^′^ *be storage distributions such that* *δ*^′^≼ *δ.* *Then* $\xi (A_{\delta ^{\prime }},C_{\delta ^{\prime }}) \le \xi (A_{\delta },C_{\delta })$*.*

A key contribution of [11] is the concept of *storage dependencies* and we refer the reader to [11] for the precise definition. Analysis of the self-timed execution of (*A*_*δ*_,*C*_*δ*_) is used to compute a set *Dep*_*δ*_ ⊆ *C*_*buf*_ of buffered channels that have a storage dependency. The throughput of a CSDF graph cannot be increased without increasing the capacity of at least one such a channel. In Section 2, we have sketched how this can be used to reduce the search space (cutting off the area filled with the pattern in Fig. 1). The following lemma and corollary formalize this.

### **Lemma 2**

[11] *Let* (*A*,*C*) *be a CSDF graph and let* *δ* *and* *δ*^′^ *be a storage distributions such that* *δ* ≼ *δ*^′^ *and* $\xi (A_{\delta }, C_{\delta }) < \xi (A_{\delta }^{\prime }, C_{\delta }^{\prime })$*.* *Then there is a channel* *c* ∈*Dep*_*δ*_ *such that* *δ*(*c*) < *δ*^′^(*c*)*.*

The following corollaries follow from these lemmas. The first one states that increasing buffers that have no storage dependency does not increase the throughput.

### **Corollary 1**

*Let* (*A*,*C*) *be a CSDF graph and let* *δ* *be a storage distribution. For every storage distribution* *δ*^′^ *holds: if for all* *c* ∈*Dep*_*δ*_ *we have that* *δ*^′^(*c*) ≤ *δ*(*c*)*,* *then* $\xi (A_{\delta ^{\prime }}, C_{\delta ^{\prime }}) \le \xi (A_{\delta }, C_{\delta })$*.*

### Proof

Consider a storage distribution *δ*^′^ with *δ*^′^(*c*) ≤ *δ*(*c*) for all *c* ∈*Dep*_*δ*_, let *t* = *ξ*(*A*_*δ*_,*C*_*δ*_), and let $t^{\prime } = \xi (A_{\delta ^{\prime }}, C_{\delta ^{\prime }})$. Assume that *t*^′^ > *t*. We define

$$\delta^{\prime\prime}(c) = \left\{\begin{array}{llllll} \delta(c) & {if}c \in \mathit{Dep}_{\delta} \\ \mathit{max}(\delta(c), \delta^{\prime}(c)) & otherwise \end{array}\right. $$

Let $t^{\prime \prime } = \xi (A_{\delta ^{\prime \prime }}, C_{\delta ^{\prime \prime }})$. We have that *δ*^′^≼ *δ*^″^, and therefore by Lemma 1 that *t*^′^≤ *t*^″^, and thus *t* < *t*^″^. Furthermore, we have that *δ* ≼ *δ*^″^. We can then apply Lemma 2 to conclude that there is a channel *c* ∈*Dep*_*δ*_ such that *δ*(*c*) < *δ*^″^(*c*). This contradicts the definition of *δ*^″^, and therefore we conclude that *t*^′^≯*t*. □

The second corollary informally states that if we have an infeasible storage distribution without buffered channels that have a storage dependency, then no feasible storage distribution exists.

### **Corollary 2**

*Let* (*A*,*C*) *be a CSDF graph and let* *δ* *be an infeasible storage distribution. If* *Dep*_*δ*_ = *∅,* *then no feasible storage distribution exists.*

### Proof

Suppose that a feasible storage distribution *δ*^′^ exists, which necessarily has a greater throughput than *δ*. Define *δ*^″^ as *δ*^″^(*c*) = *max*(*δ*(*c*),*δ*^′^(*c*)) for all *c* ∈ *C*_*buf*_. Then *δ*^′^≼ *δ*^″^ and therefore $\xi (A_{\delta ^{\prime }}, C_{\delta ^{\prime }}) \le \xi (A_{\delta ^{\prime \prime }}, C_{\delta ^{\prime \prime }})$ by Lemma 1. Thus, $\xi (A_{\delta }, C_{\delta }) < \xi (A_{\delta ^{\prime \prime }}, C_{\delta ^{\prime \prime }})$. We also have that *δ* ≼ *δ*^″^ and therefore we can apply Lemma 2 to conclude that there must be a channel *c* ∈*Dep*_*δ*_ such that *δ*(*c*) < *δ*^″^(*c*). This contradicts that *Dep*_*δ*_ = *∅*. □

In the remainder of this article, we assume that we have access to a CSDF analysis function *analyze* that, given a CSDF graph (*A*,*C*), a set of buffered channels *C*_*buf*_ ⊆ *C* and a storage distribution *δ* for *C*_*buf*_ returns a tuple (*ξ*(*A*_*δ*_,*C*_*δ*_),*Dep*_*δ*_) where *Dep*_*δ*_ ⊆ *C*_*buf*_ is the set of channels with a storage dependency in (*A*_*δ*_,*C*_*δ*_). The problem that we consider is the following:

### **Definition 3** (Optimization problem)

Given are a CSDF graph (*A*,*C*), buffered channels *C*_*buf*_ ⊆ *C*, a throughput constraint *tc*, and a cost function $w : C_{\mathit {buf}} \to \mathbb {N}$. The buffer optimization problem is to find a feasible storage distribution *δ* such that for any other feasible storage distribution *δ*^′^ holds that |*δ*|≤|*δ*^′^|.

Consider the CSDF graph (*A*,*C*) from Fig. 2 again. It has throughput 1.04 ⋅ 10^− 3^, and we use this as the throughput constraint for (*A*_*δ*_,*C*_*δ*_) shown in Fig. 3. Then *δ* = {*c*_1_↦1,*c*_2_↦4,*c*_3_↦8,*c*_4_↦14,*c*_5_↦5} has a throughput of 9.19 ⋅ 10^− 4^ and thus is not feasible. The causal dependency analysis gives us that *Dep*_*δ*_ = {*c*_1_,*c*_2_,*c*_3_,*c*_5_}. From this we can conclude that every buffer valuation {*c*_1_↦1,*c*_2_↦4,*c*_3_↦8,*c*_4_↦*x*,*c*_5_↦5} with $x \in \mathbb {N}_{0,\infty }$ is not feasible. That is, increasing only buffer *c*_4_ in size and none of the other buffers, will not lead to a better throughput. We can use this to reduce the search space as explained in Section 2.

In the next section, we formally explain the framework that we use to approach the optimization problem of Definition 3.

## Monotonic Optimization

We assume the problem setting of Definition 3 and let *d* = |*C*_*buf*_|. A storage distribution *δ* is represented by a point *x* = (*x*_1_,*x*_2_,…,*x*_*d*_) in $\mathbb {N}_{0,\infty }^{d}$ given a bijection *index* : *C*_*buf*_ →{1,2,…,*d*} as follows: *x*_*index*(*c*)_ = *δ*(*c*) for all *c* ∈ *C*_*buf*_. The cost of *x*, denoted by |*x*| is defined as |*δ*|. We say that an *x* is feasible if and only if *δ* is feasible. We use the abbreviation *x*[*i* ← *v*] for the point (*x*_1_,*x*_2_,…,*x*_*i*− 1_,*v*,*x*_*i*+ 1_,…,*x*_*d*_), i.e., the *i*-th element of *x* is replaced by *v*. In the remainder of this article, we assume that the sets *D*, *E*, *K*, *S*, and *U* all are subsets of $\mathbb {N}_{0,\infty }^{d}$, and that *k*, *s*, *u*, *q*, *x*, *x*^′^, *y*, *y*^′^, *z*, and *z*^′^ all are elements of $\mathbb {N}_{0,\infty }^{d}$. The set complement operation is assumed to act with respect to the universe $\mathbb {N}_{0,\infty }^{d}$, i.e., $\overline {U} = \mathbb {N}_{0,\infty }^{d} \setminus U$.

The forward (+) and backward (−) cones of *x* and their strict versions (+ +, =) are defined as follows:

1 $$ \begin{array}{lcl} \qquad {x}^{+} & = &\{ {x^{\prime}} | \forall_{1 \le i \le d} x_{i}^{\prime} \ge x_{i} \} \\ \qquad {x}^{++} & = &\{ {x^{\prime}} | \forall_{1 \le i \le d} x_{i}^{\prime} > x_{i} \} \\ \qquad {x}^{-} & = &\{ {x^{\prime}} | \forall_{1 \le i \le d} x_{i}^{\prime} \le x_{i} \} \\ \qquad {x}^= & = &\{ {x^{\prime}} | \forall_{1 \le i \le d} x_{i}^{\prime} < x_{i} \} \end{array} $$

If *U* is closed under −, then its complement is closed under +, and vice versa:

2 $$ \begin{array}{lcl} \qquad \overline{U^{-}}^{+} &=& \overline{U^{-}} \\ \qquad \overline{U^=}^{+} &=& \overline{U^=} \\ \qquad \overline{U^{+}}^{-} &=& \overline{U^{+}} \\ \qquad \overline{U^{++}}^{-} &=& \overline{U^{++}} \end{array} $$

The backward (forward) cone of some set *U* is the union of the backward (forward) cones of the elements of *U*. A point *x* ∈ *U* is *maximal* in *U* if and only if for all *y* ∈ *U*,*y*≠*x* holds that *x*∉*y*^−^. Similarly, *x* ∈ *U* is *minimal* if and only if for all *y* ∈ *U*,*y*≠*x* holds that *x*∉*y*^+^. We use this definition for the maximal elements of sets as follows: *max*(*U*) = {*x* ∈ *U*|*x* is maximal in *U*}, and *min*(*U*) = {*x* ∈ *U*|*x* is minimal in *U*}. A set *U* is *maximal* if and only if *max*(*U*) = *U* and *minimal* if and only if *min*(*U*) = *U*. The cones of a finite set *U* can be represented by a unique subset of itself containing only maximal or minimal elements:

3 $$ \begin{array}{lcl} \qquad U^{+} & = & \mathit{min}(U)^{+} \\ \qquad U^{-} & = & \mathit{max}(U)^{-} \\ \qquad U^{++} & = & \mathit{min}(U)^{++} \\ \qquad U^= & = & \mathit{max}(U)^= \end{array} $$

In some other contexts, these sets of minimal or maximal points are called Pareto points. In Fig. 1, for instance, the backward cone of the set of maximal points *U* = {*u*_0_,*u*_1_,*u*_2_,*u*_3_} is shown, as well as the forward cone of the set of minimal points *S* = {*s*_0_,*s*_1_,*s*_2_}. Note that the strict cones exclude the points on the boundaries, and that the knee points of *U*, {*k*_0_,…,*k*_4_}, are elements of *U*^−^. In fact, the knee points can be regarded as the duals of the points in *max*(*U*). The following definition is an alternative characterization of knee points as originally introduced in [7].

### **Definition 4** (Knee points)

The set of points (knees) of a finite set $U \subseteq \mathbb {N}_{0,\infty }^{d}$, denoted by *knee*(*U*) is the set $\mathit {knee}(U) = \mathit {min}(\overline { U^=} )$.

The following corollary states an equivalent characterization of knee points, which we use further below.

### **Corollary 3**

*K* = *knee*(*U*) *if and only if* *K* *is minimal and* $K^{+} = \overline {U^=}$*,* *i.e.,* $K^{+} \cup U^= = \mathbb {N}_{0,\infty }^{d} \land K^{+} \cap U^= = \emptyset $*.*

### Proof

(⇒) Straightforward with Eqs. 2 and 3.
(⇐) We have that $K^{+} = \overline {U^=}$. Thus, $\mathit {min}(K^{+}) = \mathit {min}(\overline {U^=})$. Because clearly *min*(*K*^+^) = *min*(*K*), we have by the assumption that *K* is minimal that *min*(*K*^+^) = *K* and thus $K = \mathit {min}(\overline {U^=}) =\mathit {knee}(U)$.

□

The next corollary states that under a specific condition, the union of the strict forward cones of the knees *K* is equal to the complement of the union of the backward cones of the unsat points *U*. The extra condition on *U* is needed because the hyperplane lower boundaries (i.e., the points in which at least one dimension equals 0) by definition are not part of *K*^++^ and hence should be included in *U*^−^. This extra condition is true for at least every set *U* for which holds that {*∞*[*k* ← 0]|1 ≤ *k* ≤ *d*}⊆ *U*^−^. In the context of buffer sizing this makes perfect sense, as it models the situation in which storage distributions that have buffers of size 0 are infeasible.

### **Corollary 4**

*Let* *K* = *knee*(*U*) *and let* *U* *be such that for every point* *x*∉*U*^−^*,there is some point* *y* ∈ *U*^−^ *such that* *y* ∈ *x*^=^*.* *Then* $K^{++} = \overline {U^{-}}$*.*

### Proof

($K^{++} \subseteq \overline {U^{-}}$) Let *x* ∈ *K*^++^. We need to show that *x*∉*U*^−^. Then by definition of *K*, $x \in \overline {U^=}^{++}$. Hence, there is some $y \in \overline {U^=}$ such that *y* ∈ *x*^=^. We have that $y \in \overline {U^=}$, so *y*∉*U*^=^ and therefore, for any *u* ∈ *U*, *y*∉*u*^=^ (1). Assume towards a contradiction that there is some *z* ∈ *U* such that *x* ∈ *z*^−^. From *y* ∈ *x*^=^ and *x* ∈ *z*^−^ it follows that *y* ∈ *z*^=^, which contradicts (1).

($\overline {U^{-}} \subseteq K^{++}$) Let $x \in \overline {U^{-}}$, i.e. *x*∉*U*^−^. We need to show now that *x* ∈ *K*^++^, i.e., that $x \in \overline {U^=}^{++}$. Let *z*_0_ be such that *z*_0_ ∈ *U*^−^ and *z*_0_ ∈ *x*^=^. Here we use the additional assumption to ensure it exists. If *z*_0_∉*U*^=^, we have some *z*∉*U*^=^ with *z* ∈ *x*^=^, thus $x \in \overline {U^=}^{++}$ and we are done. Otherwise, *z*_0_ ∈ *U*^=^, *x*∉*U*^−^ and *z*_0_ ∈ *x*^=^. Hence, there must be some *z*_1_ such that *z*_1_≠*z*_0_, $z_{0} \in z_{1}^{-}$, *z*_1_ ∈ *U*^−^, and *z*_1_ ∈ *x*^=^. Again, if *z*_1_∉*U*^=^ we are done. Otherwise we continue similarly with *z*_2_, *z*_3_, etcetera. Eventually, we must find *z*_*k*_ such that *z*_*k*_∉*U*^=^, because |*x*|−|*z*_*k*_| decreases in every step and cannot go negative. □

Finally, the following corollary formalizes that the knee points are part of the backward cone of the generating set (see, e.g., Fig. 1).

### **Corollary 5**

*Let* *K* = *knee*(*U*)*.* *If* *U*≠*∅,* *then* *K* ⊆ *U*^−^*.*

### Proof

Suppose that *U*≠*∅* and *K*⫅̸*U*^−^. Then we have that ¬∀_*k*∈*K*_∃_*u*∈*U*_*k* ∈ *u*^−^. I.e., ∃_*k*∈*K*_∀_*u*∈*U*_*k*∉*u*^−^. Consider such a *k*. We distinguish two cases. First, *k* = 0^*d*^. Because we have assumed that *U*≠*∅*, there is at least one *u* ∈ *U*, and clearly *k* ∈ *u*^−^, which is a contradiction. Hence, *K* ⊆ *U*^−^. Second, *k*≠ 0^*d*^. Define the point *x* such that *x*_*i*_ = *max*(*k*_*i*_ − 1,0) for 1 ≤ *i* ≤ *d*. In that case, *x*≠*k* and clearly *x*∉*k*^+^ and also *x*∉*K*^+^ because *K* consists of minimal points. Furthermore, *x*∉*u*^−^ since *k*∉*u*^−^ for all *u* ∈ *U* by assumption. Therefore also *x*∉*U*^=^. Thus, we have that *x*∉*K*^+^ and *x*∉*U*^=^, which constradicts Corollary 3. Hence, *K* ⊆ *U*^−^. □

**Figure 4:**
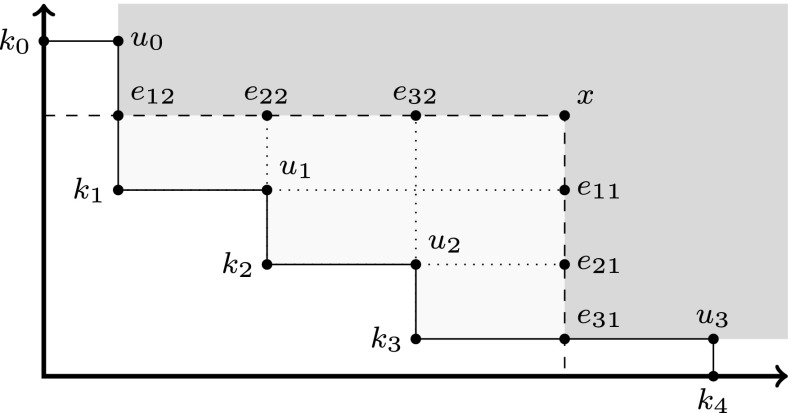
An example of knee generation.

As our algorithm sketched in Section 2 progresses, it can happen that a point *x* that just has been analyzed is infeasible. This has impact on the existing knee points of the infeasible set *U* that are in the backward cone of *x*. Figure 4 shows an example of such a situation with *U* = {*u*_0_,…,*u*_3_}. The knee points *k*_1_, *k*_2_ and *k*_3_ are in *x*^−^. Adding *x* to the infeasible set *U* has the consequence that *k*_1_, *k*_2_ and *k*_3_ are not knee points anymore. A point *k* in *x*^−^ has *d* extensions to *x*, namely the points {*k*[*i* ← *x*_*i*_]|1 ≤ *i* ≤ *d*} where each time the value of one dimension of *k* is replaced by the corresponding value of *x*. The knee points *k*_1_, *k*_2_ and *k*_3_ in Fig. 4 are replaced by the points *e*_12_ and *e*_31_, which are the minimal points of the extensions of the knees to *x*.

This is formalized by the following two lemmas and Theorem 1 on knee generation.

### **Lemma 3** (Extension completeness)

*If* *k* ∈ *x*^−^*,* *then* *k*^+^ ∖ *x*^=^ = {*k*[*i* ← *x*_*i*_]|1 ≤ *i* ≤ *d*}^+^*.*

### Proof

First, let *z* ∈{*k*[*i* ← *x*_*i*_]|1 ≤ *i* ≤ *d*}^+^. Then *z* ∈ *k*[*i* ← *x*_*i*_]^+^ for some dimension *i*. We let *q* = *k*[*i* ← *x*_*i*_] and thus have *z* ∈ *q*^+^. By definition we have *z*_*j*_ ≥ *q*_*j*_ for all 1 ≤ *j* ≤ *d*. We also have that *q*_*j*_ ≥ *k*_*j*_ for 1 ≤ *j*≠*i* ≤ *d* and *q*_*i*_ ≥ *x*_*i*_ ≥ *k*_*i*_. Thus, *z*_*j*_ ≥ *k*_*j*_ which is to say that *z* ∈ *k*^+^, but also *z*_*i*_ ≥ *x*_*i*_, which implies that *z*∉*x*^=^.

Second, let *z* ∈ *k*^+^ ∖ *x*^=^. This means that *z*_*j*_ ≥ *k*_*j*_ for all 1 ≤ *j* ≤ *d* and some *i* exists such that *z*_*i*_ ≥ *x*_*i*_. Let *q* = *k*[*i* ← *x*_*i*_]. Then we have *q*_*j*_ = *k*_*j*_ for all 1 ≤ *j*≠*i* ≤ *d* and *q*_*i*_ = *x*_*i*_ ≥ *k*_*i*_. Thus, *z*_*j*_ ≥ *q*_*j*_ for 1 ≤ *j* ≤ *d*, and *z* ∈ *q*^+^. Therefore, *z* ∈{*k*[*i* ← *x*_*i*_]|1 ≤ *i* ≤ *d*}^+^. □

The second lemma is a generalization of Lemma 3 and is the basis for our knee computation method. It formalizes (and generalizes to an arbitrary number of dimensions) the situation sketched in Fig. 4.

### **Lemma 4**

*Consider a point* *x* *and a set* *K* *such that* *x* ∈ *K*^+^*.* *Then* *K*^+^ ∖ *x*^=^ = ((*K* ∖ *D*) ∪*min*(*E*))^+^ *where* *D* = *K* ∩ *x*^−^ *and* *E* = {*y*[*i* ← *x*_*i*_]|*y* ∈ *D* ∧ 1 ≤ *i* ≤ *d*}*.*

### Proof

Note that this proof treats the term *min*(*E*)^+^ first as *E*^+^ and as a last step reasons why this is valid.

First suppose that *z* ∈ *K*^+^ ∖ *x*^=^. Then *z* ∈ *k*^+^ for some *k* ∈ *K*. We distinguish two cases: (a) *k*∉*x*^−^, and (b) *k* ∈ *x*^−^. In case (a) *k* is not removed from *K* through *D*, thus *k* ∈ (*K* ∖ *D*) ∪ *E*. Therefore, *z* ∈ ((*K* ∖ *D*) ∪ *E*)^+^. In case (b) *k* is removed as part of *D*. However, the extensions of *D* are added through set *E*, and by Lemma 3 we know that *z* ∈ ((*K* ∖ *D*) ∪ *E*)^+^.

Second, suppose that *z* ∈ ((*K* ∖ *D*) ∪ *E*)^+^. Then (a) some *k* ∈ *K* ∖ *D* exists such that *z* ∈ *k*^+^, or (b) some *k* ∈ *K* and *i* exist such that *z* ∈ *k*[*i* ← *x*_*i*_]^+^. In case (a) we thus have that *z* ∈ *k*^+^, *k* ∈ *K* and *k*∉*x*^−^ and thus *z*∉*x*^−^. Therefore, *z* ∈ *K*^+^ ∖ *x*^=^. In case (b) we have that *z* ∈ *k*[*i* ← *x*_*i*_]^+^, *k* ∈ *K* and *k* ∈ *x*^−^. Clearly also *z* ∈ *k*^+^ because *x*_*i*_ ≥ *k*_*i*_ and thus *z* ∈ *K*^+^. Furthermore, *z*∉*x*^=^ because *z*_*i*_ ≥ *x*_*i*_.

Now we have proven that *K*^+^ ∖ *x*^=^ = ((*K* ∖ *D*) ∪ *E*)^+^. It is clear that $(U_{1} \cup U_{2})^{+} = U_{1}^{+} \cup U_{2}^{+}$, and the combination with *min*(*E*)^+^ = *E*^+^ yields the desired result. □

The following theorem is fundamental to our method as it provides a means to compute knee points efficiently.

### **Theorem 1** (Knee generation)

*Consider a set* *U* *and let* *K* = *knee*(*U*)*.* *Then* *K*^′^ = *knee*(*U* ∪{*x*}) *can be computed as follows:*

- *if* *x* ∈ *U*^−^*,then* *K*^′^ = *K,and otherwise:*
- *K*^′^ = (*K* ∖ *D*) ∪*min*(*E*) *where* *D* = *K* ∩ *x*^−^ *and* *E* = {*y*[*i* ← *x*_*i*_]|*y* ∈ *D* ∧ 1 ≤ *i* ≤ *d*}

### Proof

The case for *x* ∈ *U*^−^ follows straightforwardly because then (*U* ∪{*x*})^−^ = *U*^−^. Now consider the case *x*∉*U*^−^. Using Corollary 3, we have to prove that:

1. *K*^′+^ ∩ (*U* ∪{*x*})^=^ = *∅*,
2. $K^{\prime +} \cup (U \cup \{{x} \})^= = \mathbb {N}^{d}_{0, \infty }$, and
3. *K*^′^ = *min*(*K*^′^).

Item 1 reduces to ((*K* ∖ *D*) ∪*min*(*E*))^+^ ∩ (*U* ∪{*x*})^=^ = *∅*. From Lemma 4, we have that ((*K* ∖ *D*) ∪*min*(*E*))^+^ = *K*^+^ ∖ *x*^=^ and we thus have to show that:

$$\begin{array}{ll} (K^{+} \setminus x^=) \cap (U \cup \{ x \} )^= = \emptyset & \Leftrightarrow \\ (K^{+} \setminus x^=) \cap (U^= \cup x^=) = \emptyset & \Leftrightarrow \\ (K^{+} \setminus x^=) \cap U^= = \emptyset \land (K^{+} \setminus x^=) \cap x^= = \emptyset \end{array} $$

From our assumption that *K* = *knee*(*U*), we have via Corollary 3 that *K*^+^ ∩ *U*^=^ = *∅*, which proves the first part of the conjunction. The second part is straightforward from definitions of set operations.

For item 2, we have to show – using the same reduction as above – that $(K^{+} \setminus {x}^=) \cup (U \cup \{{x} \})^= = \mathbb {N}^{d}_{0, \infty }$. Using our assumption that *K* = *knee*(*U*), we can derive in a similar way as above that this indeed is the case.

For item 3, notice that *K* ∖ *D* is minimal because *K* is minimal. Furthermore, *min*(*E*) is minimal by definition. Note that for *e* ∈*min*(*E*) holds that *e* ∈ *x*^−^. Therefore, such an extension *e* does not have a point in *K* ∖ *D* in its backward cone, which is to say that no point in *K* ∖ *D* has *e* in its forward cone. Furthermore, no extension *e* ∈*min*(*E*) has a point in *K* ∖ *D* in its forward cone, because then *K* would not have been minimal. Therefore, *K*^′^ is minimal: *K*^′^ = *min*(*K*^′^). □

The following theorem states that knees of a set of infeasible storage distributions give a (non-tight) lower bound on the cost of a feasible storage distribution.

### **Theorem 2** (Lower bound feasible cost)

*Consider a set* *U* *of infeasible points such that* {*∞*^*d*^[*k* ← 0]|1 ≤ *k* ≤ *d*}⊆ *U*^−^ *and a feasible point* *x.* *Then* |*x*|≥*min*{|*k* + 1^*d*^||*k* ∈*knee*(*U*)}*.*

### Proof

Let *K* = *knee*(*U*). First, we have that for every point *x*∉*U*^−^ holds that every buffer has at least size 1, because we assume that {*∞*^*d*^[*k* ← 0]|1 ≤ *k* ≤ *d*}⊆ *U*^−^. We can apply Corollary 4 and have that $K^{++} = \overline {U^{-}}$.

Every point in *U*^−^ is infeasible by Lemma 1 and our representation of the storage distributions. Thus, a feasible point is part of $\overline {U^{-}}$, which thus equals *K*^++^. Hence, there is some knee *k* ∈ *K* such that *k* ∈ *x*^=^ and |*x*|≥|*k* + 1^*d*^|. Thus |*x*|≥*min*{|*k* + 1^*d*^||*k* ∈*knee*(*U*)}. □

In the next section, we apply the mechanism explained in this section to the optimization problem.

## Optimization Algorithm

Algorithm 1 solves the minimization problem of Definition 3. There are four local variables: the set *U* contains infeasible points, *K* contains the knees of *U*, *x* is the point that represents the storage distribution that is analyzed, and *S* contains the feasible points (also see Fig. 1). A key invariant throughout the algorithm is that *K* = *knee*(*U*) holds. 
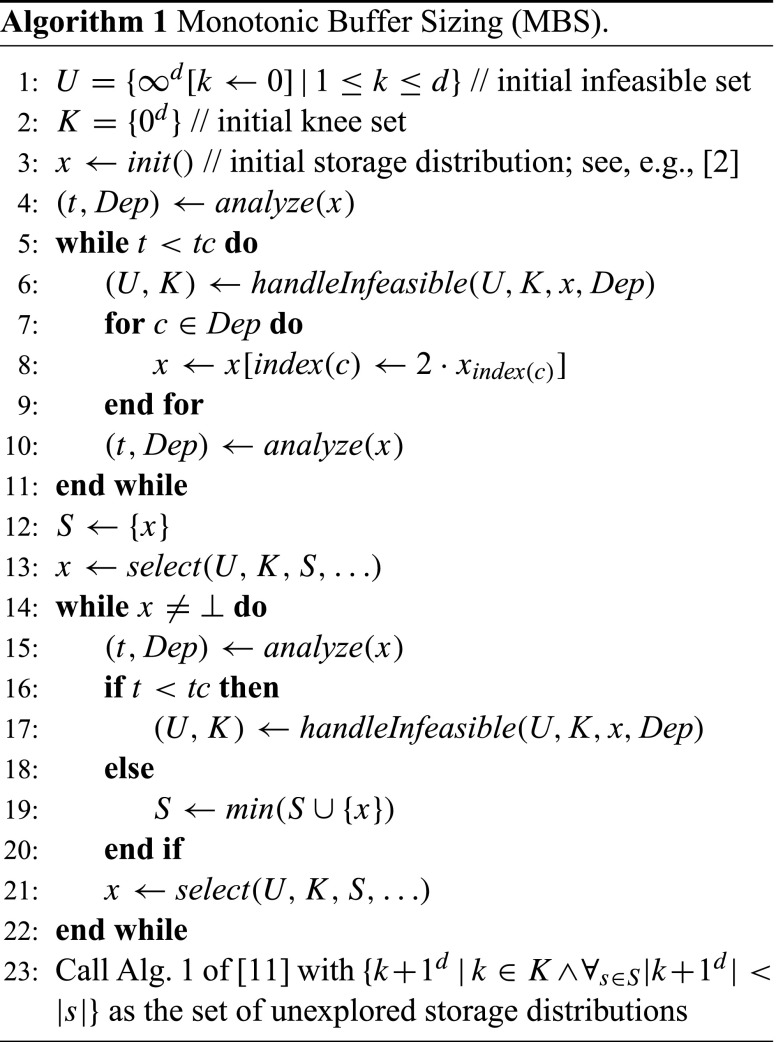
 
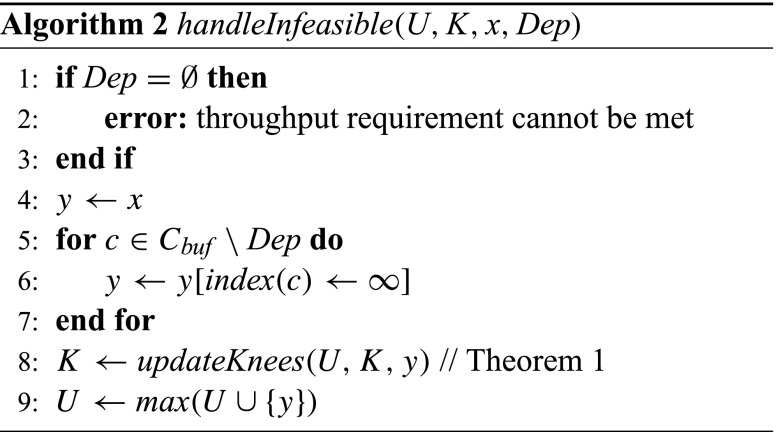

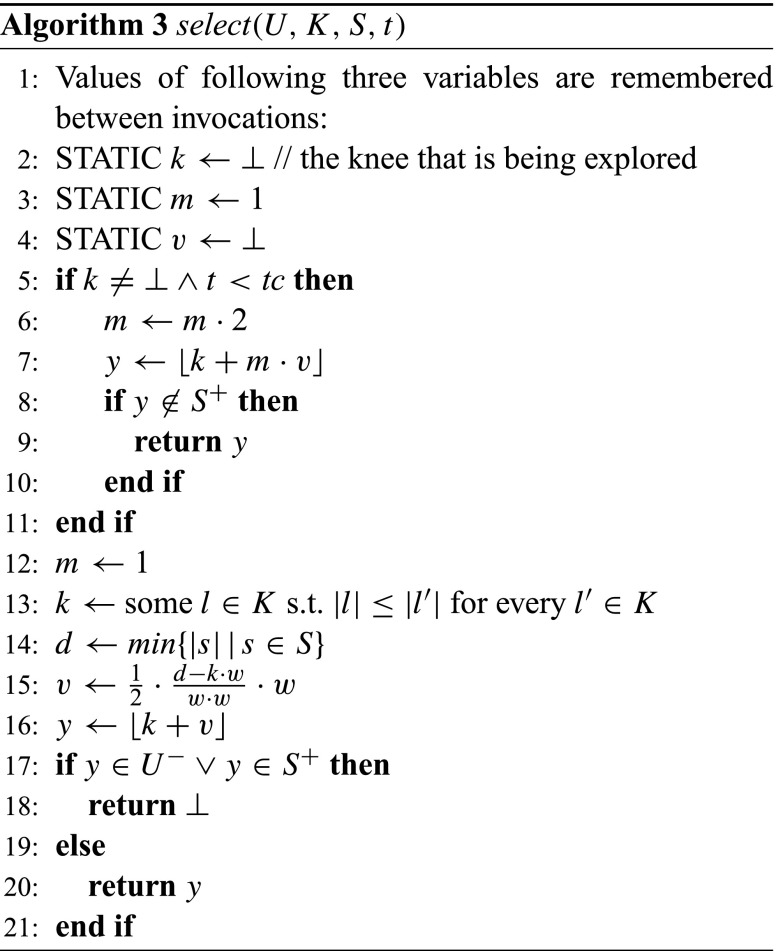




The algorithm consists of three phases. First, lines 1–11 form the initialization phase in which a first feasible solution is created, starting from the initial storage distribution that gives each buffer a minimal necessary size for deadlock-free execution (see, e.g., [2]). The while-loop iteratively doubles the buffer sizes of the buffers with a storage dependency until it finds a feasible solution. The *handleInfeasible* function, which is defined in Algorithm 2, updates the set of infeasible points *U* and the knees *K* for every infeasible point that is encountered. Note that this function reports an error if an infeasible point is encountered with no storage dependencies, which implies that there is no feasible storage distribution. Also note that lines 5 – 7 of *handleInfeasible* apply the additional pruning of the search space using the causal dependency information. This is formalized in the following lemma.

### **Lemma 5**

*Let* *x* *be* *infeasible and let* *Dep* *be* *the storage dependencies. Define* *y* = (*y*_1_,*y*_2_,…,*y*_*d*_) *as* *follows:*

$$y_{i} = \left\{\begin{array}{lllllll} \infty & if\mathit{index}^{-1}(i) \not\in \mathit{Dep} \\ x_{i} & otherwise \end{array}\right. $$

for all 1 ≤ *i* ≤ *d*. Then every point in *y*^−^ is also infeasible.

### Proof

Let *x* represent the infeasible storage distribution *δ* and consider a storage distribution $\delta ^{\prime }$ that is represented by a point in *y*^−^. By definition, $\delta ^{\prime }(c) \le \delta (c)$ for all *c* ∈*Dep*. Thus, by Corollary 1 we know that the throughput for $\delta ^{\prime }$ is not greater than the throughput for *δ*. Therefore, $\delta ^{\prime }$ is also infeasible. $\ \Box $

The second phase of Algorithm 1, lines 12–22, form the optimization phase which starts after a first feasible point is found by the initialization phase. This phase iteratively chooses a new point *x* to analyze and calls *handleInfeasible* if the point is infeasible, and otherwise adds it to *S*. The selection function is flexible (hence the … notation in the list of parameters). A requirement is that implementations either return a point that is neither in *U*^−^ nor in *S*^+^, or ⊥ (in case it cannot find a good point to explore). Our current implementation, shown in Algorithm 3, selects points on the line through a knee with minimal cost, and the closest point on the cost hyperplane of the best solution so far (see Fig. 1). It starts halfway the line segment (lines 12 – 21), and doubles the distance to the knee as long as the point is infeasible and not part of *S*^+^ to prune as much of the space as possible (lines 5 – 11). When the function cannot select a point in the unexplored space between *U*^−^ and *S*^+^, it returns ⊥. This will eventually happen because we work in $\mathbb {N}^{d}_{0, \infty }$.

The third phase of Algorithm 1, the final enumeration phase, starts in line 23. It calls the algorithm from [11] with the knee points that have the potential of leading to a feasible point with a cost smaller than the cost of the best point so far. This still is necessary because, in general, a *select* function may have left some points between *U*^−^ and *S*^+^ that may give a better solution than the best one we have found so far.

### **Invariant 1**

*At lines 10 and 21 of Algorithm1 it holds that (i)* *U*^−^ *only* *contains infeasible points, (ii)* *S*^+^ *only* *contains feasible points, and (iii)* *K* = *knee*(*U*)*.*

### Proof

Straightforward using Lemma 5 and Theorem 1.

$\ \Box $

### **Theorem 3**

*Algorithm 1 solves the optimization problem of Definition* *3.*

### Proof

The initialization phase in fact is a greedy version of the algorithm in [11] that takes exponentially growing steps in the direction of the storage dependencies. Therefore, if a feasible point exists, then the initialization phase will find one. The conclusion that no feasible solution exists for an empty set of storage dependencies is valid according to Corollary 2. The optimization phase extends the sets *U*, *K* and *S* until the selection function returns ⊥. This happens eventually, because the extension part in lines 5 – 11 eventually will find that *y* ∈ *S* in which case a new point is selected in lines 12 – 21. If *U* and *S* are sufficiently close, then, due to the fact that we have a discrete search space, we cannot find a point in between. Furthermore, if we find a point in between and process it, then either *S* comes closer to *U* (in case of a feasible point), or *U* comes closer to *S* (in case of an infeasible point). Invariant 1 ensures that *U*, *K* and *S* are built in a proper way. Finally, the algorithm from [11] is invoked with the still promising knee points as a starting point. These are good starting points because any feasible point must be part of *K*^++^, and by correctness of the algorithm in [11] we thus solve the problem of Definition 3. $\ \Box $

Note that the algorithm can also be interrupted; in that case the optimization and enumeration phases are stopped or skipped, and the best result so far *x* and the maximal cost error Δ = |*x*|−*min*{|*k* + 1^*d*^||*k* ∈ *K*} are returned (see Theorem 2). This interruption logic is not shown in Algorithm 1 for readability.

## Experimental Evaluation

We compare with the state-of-the-art approach of [11] that computes the full buffer-size – throughput trade-off space. Since the optimization problem of this article (see Definition 3) is a slightly more restricted problem, [11] can be used to solve it. In this section, we compare the approaches, because no other reference algorithm exists. We therefore set the throughput constraint to the throughput of the self-timed execution of the graph, which is the highest throughput possible. The approach of [11] terminates as soon as it has analyzed the storage distributions up to and including this self-timed throughput. Earlier results in the algorithm on trade-off points with lower throughput are needed for this, so [11] needs all earlier computations to reach the final trade-off point of the self-timed throughput. This makes the approaches comparable for the case in which we optimize the size of the storage distribution under the constraint that the throughput is maximal, i.e., equal to the self-timed throughput.

We use the following models from the Sdf3 website [1]: an MP3 playback application, an H.263 decoder, a sample-rate converter, and a satellite receiver. These are all SDF models (i.e., CSDF models with constant rates and execution times). The models MRF-32, MRF-64 and MRF-128 are models from a real-life image processing application, a multi-resolution filter with different input sizes, from the healthcare domain. The MRF models are all rather complex CSDF models with many different rates for a number of actors due to data dependencies. The cost function that we use gives each buffer a weight of one in each model.

Table 1 shows the results. For each model, we list whether it is an SDF model or a CSDF model, the number of actors |*A*| and the number of sized buffers |*C*_*buf*_|. For the state-of-the-art approach SGB08 [11], we then give the size of the obtained storage distribution |*δ*|, the number of throughput analysis calls and a running time for a given multiplication factor of the step size *n*. This multiplication factor is an approximation mechanism, i.e., a factor greater than one trades computation effort against accuracy of the obtained result: the obtained storage distribution may not be minimal anymore. The models for the MP3 playback application, the H.263 decoder and the multi-resolution filter are not analyzable within reasonable time with *n* = 1 (indicated by - in the table). The first two models even require many throughput calls with *n* = 10. For our approach, Monotonic Buffer Sizing (MBS; Algorithm 1), we also show the obtained storage distribution, the number of throughput analysis calls and a running time. The Δ column indicates the absolute maximal error in the storage distribution that we tolerate for the optimization process. This number is derived from the multiplication factor *n* and the model properties. For instance, *n* = 10 for the MP3 playback application allows an optimization error in the storage distribution of 18 (there are two buffers, and each buffer allows an error of at most 10 − 1 storage units assuming a step size of one; see [11]). Algorithm 1 needs 27 throughput analysis calls to obtain a solution with at most this error, and this is reported in the table.

**Table 1:** Experimental results.

					SGB08 [11]				MBS (Algorithm1)			
	SDF	CSDF	|*A*|	|*C*_*buf*_|	*n*	|*δ*|	# calls	Time (s)	Δ	|*δ*|	# calls	Time (s)
MP3 playback	✓		3	2	1	–	–	–	0	2898	34	5
					10	2907	1944724	8702	18	2906	27	4
H.263 decoder	✓		4	3	1	–	–	–	0	8006	46	13
					10	8023	1223707	10751	27	8029	40	12
Sample rate	✓		6	5	1	34	16	0	0	34	14	1
Satellite	✓		22	26	1	1544	38	2	0	1544	32	4
MRF-32		✓	21	4	1	–	–	–	0	500	106	31
					5	503	211	18	16	505	46	10
MRF-64		✓	21	4	1	–	–	–	0	985	115	272
					5	993	749	1002	16	993	57	106
MRF-128		✓	21	4	1	–	–	–	0	1962	149	4974
					10	1968	506	8144	36	1965	51	1112

To compare the performance of the approaches we primarily use the number of throughput analysis calls, and not the running time. The reason is that our prototype implementation has been written in Java and invokes an external Sdf3 executable for each throughput analysis, which has a significant overhead. The approach of SGB08 has, on the other hand, been fully integrated in a single Sdf3 executable. In both approaches the throughput analysis dominates the overall running time, and therefore we use the number of throughput analysis calls as a measure of performance to abstract from implementation details. The running times are, nevertheless, also shown in Table 1, and we expect that the values for a fully integrated MBS implementation will be smaller.

The results show that both approaches obtain the same size of the storage distribution when an optimal solution is expected (i.e, for a step size of one and a Δ of zero). When a suboptimal solution with a bounded error is accepted, then both approaches result in storage distributions of similar size, which is as expected. The MP3 playback application and the H.263 decoder models are difficult for SGB08, but easy for our approach. We believe that this is caused by explicit visitation of a large part of the search space by SGB08 to achieve the optimal throughput, whereas our approach takes large steps and skips analysis of many intermediate storage distributions. Both methods show similar performance for the models of the sample-rate converter and satellite receiver. These models differ from the MP3 playback and the H.263 decoder models in the fact that the initial storage distribution that can be calculated by a fast analysis is close to the optimal storage distribution with the required throughput. The results also show that our approach scales better than SGB08 for the rather complex CSDF models of the image-processing application from the healthcare domain.

## Conclusions

We have introduced an algorithm to optimize the storage distribution size given a throughput constraint for CSDF graphs. This algorithm is based on three ingredients: (i) the causal dependency analysis from [10, 11], (ii) principles from the area of monotonic optimization [12, 13], and (iii) the concept of knee points introduced in [7]. A useful property of our algorithm is that it can provide some feasible storage distribution and an upper bound on the size difference with an optimal feasible storage distribution any time after the initialization phase. The experimental results show that our approach is better suited for buffer minimization under a throughput constraint than (the more general) approach of [10, 11] in the sense that solutions can be obtained with fewer throughput analysis calls.

Our algorithm can in principle be applied to other models of computation and other optimization problems than buffer sizing in CSDF by removing or adapting the parts with respect to causal dependency analysis (i.e., line 23 in Algorithm 1 and lines 5 – 7 in Algorithm 2). The only requirement is that the function that defines the feasibility is monotone with respect to the optimization parameters (in our case the throughput is monotone with respect to the buffer sizes; see Lemma 1). The resulting approach then would be closely related to the generic monotonic optimization frameworks as presented in [12, 13].

## Footnotes

1 If *δ*(*c*) = *∞*, then we can model this by removing the buffer edge for channel *c*.

2 Buffer sizing for non-strongly-connected graphs can be done for the strongly-connected components.
